# 

**Published:** 1884-05

**Authors:** 


THE American Medical Association will meet in Washington, May 6th. We have received the following note from Mr. B. W. Wrenn, the General Passenger Agent of the "Kennesaw Route:"

"Delegates to the American Medical Association, which meets in Washington, D C., May 6th, can secure Round Trip Tickets *via* the Kennesaw Route, on May 3d, 4th and 5th, at $23 45, good to return within twenty days. Pullman cars leave Atlanta daily at 2.35 P M. and 3 20 P. M., for Washington without change.

B. W. WRENN,

*General Passenger and Ticket Agent.*"

THE SIMS MEMORIAL FUND.

TO THE MEDICAL PROFESSION AND OTHERS THROUGHOUT THE WORLD.

THE great achievements of Dr. J. Marion Sims call for some more lasting testimonial than obituaries and eulogies. To him medical science is indebted for much brilliant and original work, especially in gynæcological surgery. Those who have been benefited by his teachings and new operations, and such as have had the direct advantage of his personal skill, are among the first to recognize and acknowledge this debt.

To him is due the honor of giving the first strong impulse to the study of gynæcological surgery in America.

It is believed that the medical profession everywhere, the vast number of women who owe their relief from suffering directly to him, and those who realize the benefits he first made possible, will gladly unite thus to honor the man through whose original and inventive genius such blessings have been conferred upon humanity.

At the suggestion of many friends, therefore, the subjoined committee has been organized, and it is proposed that a suitable monument be erected to his memory in the City of New York.

To this end the active co-operation of the medical profession and the many other friends of Dr. Sims throughout the world, is respectfully solicited. Contributions of one dollar and upward may be forwarded to this journal which has been constituted the treasury of this fund—*The Medical Record* New York.

FORDYCE BARKER. M D., Chairman.
GEORGE F. SHRADY, M. D., Secretary.

T. Addis Emmet, M.D., New York; T. Gaillard Thomas, M D., New York; William T. Lusk, M.D., New York; William M Polk, M.D., New York; Paul F. Mundé, M D., New York; S. O. Vander Poel, M.D., New York; Frank P. Foster, M D., New York; E. S. Gaillard, M.D., New York; A. J. C. Skene, M.D., Brooklyn, N. Y.; S D. Gross, M.D., Philadelphia, Pa; Wm. Goodell, M.D., Philadelphia, Pa; J. R Chadwick, M.D., Boston, Mass.; Wm. H. Byford, M. D, Chicago, Ill.; A. R. Jackson, M.D., Chicago, Ill.; T. A. Reamy, M.D, Cincinnati, Ohio; C. D. Palmer, M D., Cincinnati, Ohio; G. J. Engleman, M.D., St. Louis, Mo.; R. Beverly Cole, M.D., San Francisco, Ca.; H. F. Campbell, M.D, Augusta, Ga.; R. B. Maury, M.D., Memphis, Tenn.; E. S. Lewis, M.D., New Orleans, La; J. T. Searcy, M.D., Tuskaloosa, Ala.; Hunter Mcguire, M.D., Richmond, Va.; S C. Busey, M.D., Washington, D. C.; H. J. Byrd, M D., Baltimore, Md.; W. J. Howard, M D., Baltimore, Md.; D. W. Yandell, M.D., Louisville, Ky; Seth C. Gordon, M.D., Portland, Me.; F. E Beckwith, M. D., New Haven, Conn.; A. W. Knox, M.D., Raleigh, N. C.; L. W. Oakley, M.D., Elizabeth, N. J.; E. A. Woodward, M.D., Brandon, Vt.; Alfred Crosby, M.D., Concord, N. H; E. S. Dunster, M.D., Ann Arbor, Mich.; A. J. Stone, M.D., St. Paul, Minn.; R. A. Kinlock, M.D., Charleston, S. C.

Other names may be added to this list from time to time. Over $2 200 paid in.

---

THE Florida State Medical Association will hold its annual meeting this year, in the city of Jacksonville, beginning Wednesday, June 4th.

### BOOKS AND PAMPHLETS RECEIVED.

Da Casta's Medical Diagnosis, Sixth Edition—J. B. Lippincott & Co, Philadelphia, 1884.

Shakespeare as a Physician—By J. Portman Chesney, M. D. J. H. Chambers & Co., Chicago, St. Louis and Atlanta, 1884.

Manual of Practical Hygiene, Parkes, Vol. II—Wm. Wood & Co., New York.

Diseases and Injuries of the Horse. Kirby—Wm. Wood & Co., New York.

Aneurism of the Femoral Artery and a knife-wound of the Intestines--W O. Roberts, M. D., Louisville.

Transactions of the Massachusetts Medico-Legal Society, Vol. 1, No. 6, 1883—Riverside Press, Cambridge, 1884.

The Proceedings of the Naval Medical Society, Vol. 1, No. 6—Judd & Detweiler, Washington, 1884.

Catalogue of the Young Men's Library of Atlanta—Jas. P. Harrison & Co., Atlanta, 1884.

Epileptic Insanity—By Philip Zeuner, A. M. M. D., Cincinnati.

The Effect of the Inundations of the Mississippi River upon Health--By R. H. Day, Baton Rogue, La.

Annual Report of the Presbyterian Eye, Ear and Throat Charity Hospital—Baltimore.

Notes on the Opium Habit--By Asa P. Meylert, M. D., G. P. Putnam's Sons, New York, 1884.

### OUR ADVERTISERS.

We present to our readers in this number of THE JOURNAL, a number of new advertisements which we hope they will read. In corresponding with these firms they will confer a special favor upon the publishers if they will mention THE JOUBNAL.

Aloe Hernstein & Co., St. Louis. This well established instrument house has for a long time been the chief instrument depot of the West, and is rapidly gaining popularity in the South, and we think deservedly so. From them can be had any instrument or surgical appliance known to the profession. They also manufacture any new instrument or appliance ordered when proper drawings of the same are sent them. They also carry a full line of batteries, saddle-bags, etc. See their advertisement and write them for a catalogue.

A. A. Mellier, St. Louis. This gentleman, besides getting up the best saddle-bag in the market, is sole proprietor and manufacturer of Tongaline, which is a combination of Tonga with the Salicyliates of Sodium, Pilocarpin and Colchicin. It is highly recommended in muscular rheumatism and nervous headaches. Read his advertisement and write to him for a sample.

Chas. O. Tyner, Atlanta. The handsomest drug store in the South is that of Mr. C. O. Tyner, at corner of Broad and Marietta streets, Atlanta. This house is first-class in every respect. Parties ordering goods by mail can rely upon Mr Tyner as being strictly honest, and upon getting just what they order.

Any rare drug that physicians may not be able to get in smaller towns, can be had by ordering of Mr. Tyner.

Doliber, Goodale & Co., Boston. These gentlemen are proprietors of the justly celebrated Mellin's Food for infants and invalids. The managing editor of THE JOURNAL has had frequent opportunities of testing its efficacy as a food for infants, and unhesitatingly recommends it. He feels safe in saying that a fair trial of it in children suffering from indigestion as a result of improper diet, will sustain him in this recommendation See their advertisement on fourth page of cover, and write them for a sample bottle, mentioning THE JOURNAL, and you will receive it without delay.

ORIGINAL COMMUNICATIONS—

NOTES—

Testis in Perineo, 147; How to take a Pill, 149; Rule for Reducing Dislocations of the Hip Joint, 157; Tubal Fœtation, 163; American Medical Association, 185; The Sims Memorial Fund, 185; Florida State Medical Association, 186; Facial Erysipelas, 191; A Toothache Remedy, 192; Death from Ether, 192; Items, 192.

### NOTICE.

Original communications solicited. When articles require illustration the engravings will be furnished by the publishers free of charge.

SUBSCRIPTION: $2.50 a year.

Subscribers will please not send us money by Postal Notes. Remit by Express, Draft, Post-office Order or Registered Letter.

Address all letters and books and make all drafts payable to

THE ATLANTA MEDICAL AND SURGICAL JOURNAL,
Care Jas. P. Harrison & Co., Atlanta, Ga.

